# A five-year review of burn injuries in Irrua

**DOI:** 10.1186/1472-6963-7-171

**Published:** 2007-10-23

**Authors:** Andrew E Dongo, Eshobo E Irekpita, Lilian O Oseghale, Charles E Ogbebor, Christopher E Iyamu, John E Onuminya

**Affiliations:** 1 Departments of Surgery, Irrua Specialist Teaching Hospital, Irrua. Edo State, Nigeria; 2 Department of Orthopaedics and Traumatology, Irrua Specialist Teaching Hospital, Irrua. Edo State, Nigeria

## Abstract

**Background:**

The management of burns remains a challenge in developing countries. Few data exist to document the extent of the problem. This study provides data from a suburban setting by documenting the epidemiology of burn injury and ascertaining outcome of management. This will help in planning strategies for prevention of burns and reducing severity of complications.

**Methods:**

A total of 72 patients admitted for burns between January 1st, 2002 and December 31st, 2006 at the Irrua specialist teaching hospital were studied retrospectively. Sources of information were the case notes and operation registers. Data extracted included demographics as well as treatment methods and outcome

**Results:**

The results revealed male to female ratio of 2.1:1. Over 50% of the injuries occurred at home. There was a seasonal variation with over 40% of injuries occurring between November and January. The commonest etiologic agent was flame burn from kerosene explosion. There were 7 deaths in the series.

**Conclusion:**

Burns are preventable. We recommend adequate supply of unadulterated petroleum products and establishment of burn centers.

## Background

Burn injuries are a common cause of mortality and morbidity in Nigeria [1]. They have now assumed alarming levels with a recurrent spate of pipeline explosions from pipeline vandalisation [2]. The harsh socioeconomic situation, absence of gainful employment coupled with greed have been cited as a cause of the upsurge in pipeline explosions [3].

Although mortality from burn injury worldwide has been on the decline [4], it still represents a significant contributor of morbidity and mortality in a rural African setting [5-7]. Developments that have helped in the reduction of mortality in the advanced world include promulgation of prevention programs [8], advances in pre-hospital care, management in intensive care units in well equipped burn centres, early excision of burn wound and skin cover [9] and use of newer skin substitutes [10]. In Nigeria and most of Africa, pre-hospital care is non existent. First aid when given involves applying a myriad of agents that may infact be harmful [11]. In addition the absence of adequate facilities and trained medical personnel for treatment and rehabilitation contributes to increasing morbidity and mortality [12]. Burn devastates the individual as well as the community. The physical and psychological scars take long to recede [13]. Management is very tedious with high direct costs as well as indirect costs from lost man hours [14]. This requires us to devote more efforts to preventive programs to reduce incidence of burns.

In our resource poor setting, at Irrua, although a teaching hospital, we neither have plastic/burn surgeons nor specialist burn nurses. Our catchments area for provision of health services includes central and northern Edo state as well as the three neighbouring states of Ondo, Kogi and Delta. The population involved is between 3–4 million people.

Our management, involves following the ABCDE protocol of resuscitation [15]. We administered occlusive dressings with honey after simple wound debridement. Pure honey is an agent, that is cheap and effective [16]. Honey is provided by the hospital pharmacy which filters honey obtained from honey farms. The Pax Benedict catholic Monastery in Ewu has now become the largest provider of honey to our hospital in the last two years. The Monastery has a Bee farm and is registered by the National Agency for food, drug administration and control Nigeria (NAFDAC). Analgaesics and tetanus prophylaxis were routinely administered on admission. Antibiotics were commenced after wound swabs. Care was supervised by the Orthopaedic surgeons until January 2005 when general surgeons became responsible for burn patients. This study provides data from a sub-urban setting and a frame work to compare future improvement in outcome.

## Methods

This is a retrospective study of all patients admitted for burns between January 1st, 2002 and December 31st, 2006. Seventy two patients with complete records were retrieved. Burn injuries in adults were admitted first into the accident and emergency ward. Children so affected were admitted to the children's emergency ward. Burn wound was estimated by the Wallace's rule of nine to determine extent. Resuscitation was carried out with Parkland's formular. However in the absence of Ringers lactate, Normal saline was substituted. After stabilization they were admitted to the surgical wards. The wards are divided into six bays of six beds each. Efforts are made to keep only burn patients in a dedicated bay but oftentimes patient load precludes this. There was no facility for Intensive care admission during the period under review. Wounds were dressed with honey impregnated gauze over sofratulle and wrapped with gamgee. Dressings were changed daily. In some cases Vaseline impregnated gauze was used in place of Sofratulle to reduce cost. Serial wound swabs for microscopy culture and sensitivity were done usually at admission, on day five and two weekly afterwards. The clinical state was the best guide to more frequent swabs. Broad spectrum parenteral antibiotics usually with ciprofloxacin and metronidazole were commenced on admission because of reasonable suspicion of contamination from various agents used as first aid and the crude means of transportation to hospital with the patients lying on their wounds in open vans. Outcome was determined at discharge, death, referral or follow-up. Data were analysed using SPSS 11 Statistical Package.

## Results

Seventy two patients with burn injury were identified. Of these 49(68%) were males and 23(31.9%) were females giving a male to female ratio of 2.1:1. (Table 1)

**Table 1:** Sex distribution on burn injuries

Sex	Number of Cases	Percentage
Male	49	68.1
Female	23	31.9

The age range of patients was from 5 months to 73 years with an average age of 26.06 (± 16.44) years. Three of the patients were below one year. Two suffered burn wound while being strapped to their mothers back. In all 17 (23.6%) patients were less than 10 years old. Two toddlers sat on boiling soup. The commonest age group involved in burn wound was between 20–29 years where there were 20(27.7%) patients. Fifty five (72%) of patients were less than 40 years of age.(Table 2).

**Table 2:** Age distribution of burn injuries

**Age**	**Male**	**Female**	**Total (%)**
0–10	11	6	17 (23.6)
11–19	7	2	9 (9.7)
20–29	13	7	20 (27.7)
30–39	9	3	12 (16.6)
40–49	6	4	10 (13.8)
50–59	3	1	4 (5.5)
60–69	1	-	1 (1.3)
70–79	1	-	1 (1.3)

**Figure 1:**
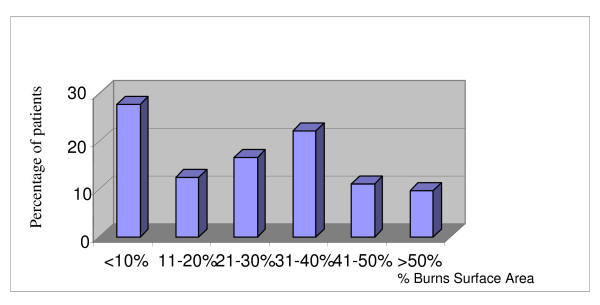
Bar chart showing distribution of burns by % burns burn surface area.

Over 90% of the burn wounds were less than 50% The percentage distribution of Burn Surface Area (BSA) is shown in Figure 1.

20 (27.7%) patients were admitted for burns involving less than 20% of their BSA. There were 7(9.7%) mortalities, five of these mortalities were in patients with over 50% BSA burns. One survivor sustained 75% flame burns.

The commonest etiological agent of burns was flame. This accounted for 44(61.1%) patients. Kerosene was slightly more than petrol burns 23(31.9%) compared to 21(29.1%). The kerosene burns resulted from explosion of lanterns and stoves during the process of refilling. Six of the petrol burns were suspected of coming from Pipe line vandalisation. Eleven were associated with road traffic accidents. Next commonest cause was scald burn in 17(23.6%) patients. Electrical burns and chemical burns were next with five (7%) and four (5%) respectively.

**Figure 2:**
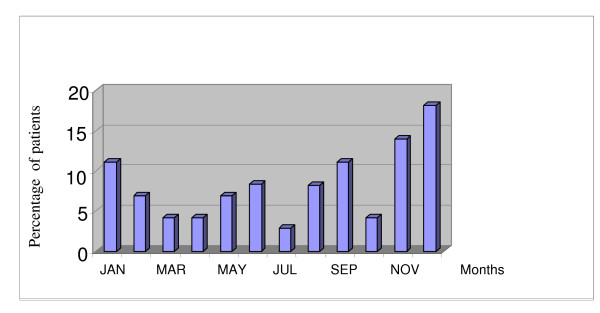
Bar chart showing monthly distribution of burns injuries.

There was a clear seasonal variation to occurrence of burn wounds as 31(43%) patients presented between November and January. December was the month with the single largest incidence.(see Figure 2).

Majority of burn injury, 40(55.5%) occurred at home. Sixteen (22.2%) patients sustained burns in the streets and Eleven (15.2%) at their work place, (Table 3).

**Table 3:** Locations where burns cases occurred

**Location**	**No (%)**
Home	40 (55.6)
Street	16 (22.2)
workplace	11(15.3)
Others	5 (6.9)

There were 7(9.7%) patients with documented wound infection. Culture of the wounds yielded Pseudomonas aeruginosa and Staphylococcus aureus infection in two patients each, while Coliform, Proteus mirabilis and Streptococcus pneumoniae were isolated in one case each. Two patients presented after discharge for contracture release, over 80% of patents were lost to follow up after one month of discharge.

## Discussion

Burn injury is an important cause of hospital admission. Age and sex are important epidemiological determinants for injuries and our study identifies the young male as being at greater risk. Many other studies in Nigeria have identified a slightly lower male to female ratio [17,18] than ours, although our ratio of 2.1:1 is less than the ratio in Singapore [19]. This is understandable because the young male is often adventurous and the bread winner for the family. This implies that apart from costs borne by the patient during admission, there is an even greater loss from the potential income lost by the bread winner [1] as a result of hospitalization and during rehabilitation. Children less than ten is the age group next commonly involved. Lack of parental supervision and awareness of surrounding have been identified as reasons for high incidence of burns in children [20]. It is noteworthy that two children suffered burns from hot soup pots that had been stored in the room. The parents lived in single room apartment known in local parlance as "face me I face you" apartments. Both had no kitchens and no stores. Improved housing conditions would diminish such risks.

Although the etiology of burn is known world wide, there are variations according to regions as our study documents flame burn as being of greater importance in the causation of burns. This finding agrees with many others from within and outside Nigeria [18,19,21]. Adulteration of petroleum products is thought to be responsible for this especially because of the perennial scarcity of petroleum products in Nigeria [21]. Some traders dubiously mix kerosene with a cheaper condensate to sell to make quick gains, but it may also be from carelessness of people using the same storage cans for kerosene and petrol or transporting kerosene in tankers previously used for petrol. There was only one case of flame burn from gas explosion, perhaps underlying the fact that gas use is associated with affluence and most victims of burns are in the lower socioeconomic strata of society. There were 4 cases of acid burns. One of this was accidental in a battery charger, a clear occupational hazard but 3 others were clearly from assault and attempted homicide. One resulted in death. The victims were suspected of involvement in cult activities although they denied it. There was also a significant contribution from electrical injury 5(7%) patients. These last two etiologies appear higher that what other studies have documented in Nigeria [18,21,22] and may become more significant in the future.

It is clear from this study that burn injury is commonest during the dry season. The reason is partly because dryness aids combustion and usually the Harmattan season in December is very cold and increases the desire to provide warmth by lighting stoves and boiling water. A study from Maiduguri which experiences extreme of harmattan has confirmed this trend [22]. This study is at variance with that by Momoh et al [18] who found burn injuries commonest in the rainy season. Over 80% of the burn injuries arrived in hospital within 24 hours with at least 60% arriving within 6 hours. Despite widespread recommendation of use of running water to wash burn wounds to prevent further damage [23,24], there was little proof of this first aid from our study. It is conceivable that most patients are ignorant of what of first aid to administer, but in any case, the environs lack running water. The greater the burn injury the sooner they arrived hospital. The reason is partly because of the refusal of primary care centres to intervene in such extensive injuries. This fact in addition to our early commencement of antibiotics partly explains why we had fewer wound infection. There were seven documented cases of infection from our study. The common organisms isolated were Staphylococcus aureus and Pseudomonas. Although our infection rate is lower than most other Nigerian studies [22,25], the retrospective nature of our study and early antibiotic use call for caution in interpreting this result. We had seven mortalities, mainly in patients who sustained greater than 50% burns. Two survivors had 50 and 75% burns respectively and survived inspite of absence of Intensive care facilities. Provision of a well equipped burn centre will reduce further such mortalities. A prospective study would be needed to follow up our patients to determine extent of post burn morbidity. This will help ascertain how adequate our care has been.

## Conclusion

We advocate health education targeted towards burn prevention. We recommend creation of employment and adequate housing. There is a need to establish a burn centre in each geopolitical zone in Nigeria

## Competing interests

The author(s) declare that they have no competing interests.

## Authors' contributions

DAE conceived the study and its design, participated in data collection and coordination as well as draft of manuscript.

IE participated in design of study and review of manuscript.

OLO, OCE, ICE assisted in data collection and review of manuscript.

OJE participated in study design, coordination and draft of manuscript.

All authors have read and approved the final version of the manuscript.

## Pre-publication history

The pre-publication history for this paper can be accessed here:



## References

[B1] Olaitan PB (2005). Burns and scalds-Epidemiology and prevention in a developing country. NJM.

[B2] Nwachukwu C, Adeyemi K, Oke B, Adetayo O, Obasola K (2006). Past pipeline explosions. The Punch.

[B3] Adeyemi K (2006). Explosions: Obasanjo expresses sadness. The Punch.

[B4] Brigham PA, Mcloughlin E (1996). Burn incidence and medical care in the United state: estimates, trends and data sources. J burn care Rehabil.

[B5] Mock CN, Adzotor E, Demo D, Conklin E, Pivara F (1995). Admissions for injury at a rural hospital in Ghana: implications for prevention in the developing world. Am J Public Health.

[B6] Kalayi GD (2006). Mortality from burns in Zaria experience from a developing economy. East Afr med J.

[B7] Gandini D (1996). Burn sequalae in Central Africa: Report on the treatment of eleven cases. Ann Burn Fire Dis.

[B8] Thompsen RM, Corrougher GJ, carrougher G (1998). Burn prevention. Burn care and therapy.

[B9] Wu XW, Herdon DN, Spies M, Sandford AP, Wolf SE (2002). Effects of delayed wound excision and grafting in severely burned children. Arch surg.

[B10] Chester DL, Pappini RPG (2004). Skin and skin substitutes in burn management. Trauma.

[B11] Olaitan PB, Iyidobi EC, Olaitan JO, Ogbonnaya IS (2004). Burns and scalds: First aid home treatment and implications at Enugu, Nigeria. Ann Burn Fire Dis.

[B12] Linares AZ, Linares HA (1990). Burn prevention: the need for a comprehensive approach. Burns.

[B13] Perry S, Difede J, Musngi G, Frances AJ, Jacobsberg L (1992). Predictors of posttraumatic stress disorder after burn injury. Am J Psychiatry.

[B14] Wibbenmayer LA, Amelon MA, Loret de Mola RM, Lewis R, Kealy GP (2003). Trash and brush burning: An underappreciated mechanism for thermal injury in a rural community. J burn care rehabil.

[B15] Hettiaratchy S, Papini (2004). ABC of burns, Initial management of burns: an Overview. BMJ.

[B16] Sabrahmanyam M (1991). Topical application of honey in treatment of burns. Br J Surg.

[B17] Adesukanmi K, Oyelami OA (1995). The pattern and outcome of burn injuries at Wesley Guild Hospital, Ilesha, Nigeria: a review of 156 cases. J Trop Med Hyg.

[B18] Momoh MI, Okugbo SU, Ohanaka EC, Ugbeye ME (2000). Causes and risk factors in burns; Experience from Benin city. Nigerian journal of surgical sciences.

[B19] Song C, Chua A (2005). Epidemiology of burn injury in Singapore from 1997–2003. Burns.

[B20] Attia AF, Sherif AA, Massoud MN, Abou-Nazel MW, Arafa MA (1997). Epidemiological and sociocultural study of burn patients in Alexandria, Egypt. East Med Health J.

[B21] Fasika OM (1997). Changing pattern of burn Epidemiology and the compliance factor in management at Ibadan. The Nigerian post graduate med J.

[B22] Dogo D, Yawe T, Na'Aya HU (1997). Burns injury in North eastern Nigeria. Nig J Surg.

[B23] Lawrence JC (1996). First aid measures for the treatment of burns and scalds. J wound care.

[B24] Nguyen OL, Gun RT, Sparnon AL, Ryan P (2002). The importance of immediate cooling in a case series of childhood burn in Vietnam. Burns.

[B25] Datubo-Brown DO, Kejeh BM (1989). Burn injuries in Port Harcourt, Nigeria. Burns.

